# Exploring the Representation of Depressive Symptoms and the Influence of Stigma in Arabic-Speaking Refugee Outpatients

**DOI:** 10.3389/fpsyt.2020.579057

**Published:** 2020-11-12

**Authors:** Nico Lindheimer, Carine Karnouk, Eric Hahn, Dana Churbaji, Laura Schilz, Diana Rayes, Malek Bajbouj, Kerem Böge

**Affiliations:** Department of Psychiatry and Psychotherapy, Campus Benjamin Franklin, Charité - Universitätsmedizin, Berlin, Germany

**Keywords:** refugee, Arabic, depression, somatic, stigma

## Abstract

The number of distressed refugees from the Arab world is relatively high in Germany and other host countries worldwide. For this specific population, substantial challenges and barriers have already been identified that hamper access to Germany's health care system. This study aims to contribute to this line of research by exploring the representation of depressive symptoms, both somatic and psychological, in order to inform clinicians about the most prevalent symptoms reported by Arabic-speaking refugee outpatients. Furthermore, this paper investigates the longstanding claim that mental health stigma fosters the expression of bodily distress. For these purposes, a total of 100 Arabic-speaking refugee outpatients, mostly Syrians, were recruited in Berlin, Germany. Somatic and psychological symptoms were assessed with the Patient Health Questionnaire (PHQ) 15 and 9, while stigma was assessed with the Brief Version of the Internalized Stigma of Mental Illness Scale (ISMI-10). Study results show that both somatic and psychological symptom severity was moderate while sleeping problems and energy loss were the most reported symptoms across both scales. Stigma was low and showed no association with somatic complaints in a multiple regression analysis, but was associated with more psychological symptoms. A principal factor extraction on the PHQ-15 items revealed five independent, somatic symptom clusters that were interpreted considering the rich poetic resources of the Arabic language. In conclusion, both somatic and psychological symptoms were commonly reported by Arabic-speaking refugees with symptoms of depression. Whereas, stigma does not seem to influence the expression of somatic symptoms, the present results provide first empirical indications for the relationship of symptom expression with the use of explanatory models and conceptualizations of mental illness within the Arabic culture and language. Future research efforts should be dedicated to enhancing our understanding of mental health care needs in this population as well as to exploring other mediators that might help explain the varying degree of somatic symptoms in depression across cultures.

## Introduction

In recent years, the number of individuals who have been forcibly displaced as a consequence of persecution, conflict, and violence around the world has risen to nearly 70 million. This number accounts for the highest total ever recorded by the United Nations High Commissioner for Refugees (1). As a result, more than 1.6 million asylum requests have been registered by the German Federal Office for Migration and Refugees (BAMF) since 2015, making Germany one of the most important host countries for refugees in the world (2, 3). In 2018, most refugees arrived from Syria (27.1%), followed by Iraq (10.0%), Nigeria (6.4%), Iran (6.3%), Turkey (6.3%), and Afghanistan (6.3%) (3). According to a representative survey of over 2,000 refugees in Germany, the main causes of flight include violent conflicts, war, prosecution, and impressment (4). Since Arabic-speaking countries currently constitute the largest number of displaced people, Arabic is considered by far the most frequently spoken native language within the refugee population in Germany (4, 5).

Exposure to traumatic events before and during migration, coupled with stressful experiences in the host environment, have been found to cause increased rates of psychological distress among refugee populations (6–9). Nonetheless, reliable epidemiological studies investigating the prevalence and course of mental illness in Germany's refugee population remain scarce (6, 10). Available tentative data, from rather small refugee samples, indicate that prevalence rates for any psychiatric disorder range between 30.5 and 95% (11–15). A recent meta-analysis estimates that the prevalence for depression in non-help-seeking Arabic-speaking refugees in Germany is 38% (95%-CI: 27–50%) and 29% (95%-CI: 21–37%) for symptoms of a post-traumatic stress disorder (PTSD) (Hoell, under review).

In light of the high need for psychiatric and psychotherapeutic treatment in this population, it is striking that refugees rarely have access to adequate and effective treatment services (16). As a contributing factor, multiple barriers seem to exist that hamper access to the German health care system for refugees and asylum seekers (10, 17). These comprise of institutional and structural barriers, such as restrictions through the Asylum-Seeker's Benefits Act and the lack of multilingual clinicians, as well as individual barriers, including lack of knowledge and language skills, shame, social, and cultural barriers (10, 17, 18). Moreover, it might be necessary to investigate whether and how experiences of trauma, war, and forced migration, coupled with a shared cultural background, translate into specific symptom representations and dysfunctions that contribute to misdiagnosis and delays in efficient and effective treatment in this population (19, 20).

Research on cultural differences in psychopathology has long focused on somatization in non-Western cultures, which can be defined as a process by which psychological distress is expressed in somatic terms (21). However, this line of research has often been driven by Western, rather stereotypical perspectives on culture, coupled with a tendency to equate culture with an ethnocultural group or merely using country of origin as a proxy (21, 22). Thus, calls for research practices that take on a more nuanced view and thereby identify the influences of specific cultural contexts and processes on psychopathology have recently raised (21). Such more sophisticated approaches have contributed to a more profound understanding of somatic symptoms in depression in cross-cultural research: In general, somatic symptoms can be considered a universal phenomenon in depressed individuals across cultures (23). Moreover, an epidemiological study with Chinese individuals in Hong Kong has shown that somatic and depressive symptoms seem to be positively correlated, which contradicts the notion that somatic symptoms are merely an immature expression of emotional distress (24). Still, an abundance of literature has found differences in the phenomenology of somatic symptoms in depression across cultural groups (19). As one potential mediator, Ma-Kellams has identified differences in somatic awareness and interoceptive accuracy across cultures and was able to link these to variations in the expression of somatic symptoms in psychopathology (25). Similarly, Ryder and colleagues showed that the relationship between culture and the presence of somatic symptoms was mediated by a tendency toward eternally oriented thinking (26). In conclusion, Kirmayer and Ryder argue that differences in the bodily expression of distress across cultures may be linked to culturally mediated modes of symptom interpretation which may be the result of stigma and available sources of help (21).

The notion that stigma might foster somatic symptom expression has often been suggested in the literature as an explanation for the observed cross-cultural variations [e.g., (21, 27)]. In general terms, mental health stigma can be understood as the negative stereotyping, biases, and discrimination faced by people with mental illness which negatively impacts the lives of affected persons in various ways (28). However, empirical evidence to support these claims is scarce and rather contradictory. Whereas, Wang et al. (29) and Rao et al. (30) found a significant association between stigma and somatization in a sample of Chinese undergraduate students and South Indian psychiatric inpatients, neither Heredia Montesinos et al. (31) nor Raguram et al. (32) found such an association in Turkish migrants or South Indian psychiatric outpatients, respectively.

In the literature on Arab mental health, various sources have suspected a causal relationship between mental health stigma and somatic symptoms (33–35). For instance, Al-Krenawi and Graham (34) attribute somatic symptom expression to a higher social acceptability of physical over psychological complaints in Arab cultures. In general, mental health stigma has been found to be highly prevalent in both Arab cultures and refugee populations (36–38). For instance, Dardas et al. (39) report that 88% of a representative sample of Jordanian adolescents have moderate to high stigma concerning depression. This in turn influences the help-seeking behavior for mental health issues, as individuals from Arab cultures fear bringing shame not only to themselves, but also to their families (40). Similarly, refugee adolescents from different countries have been shown to label mental health problems with a type of “craziness” that has to be hidden, because it negatively influences family reputation, social status and marriage prospects (41). As such, the population of Arabic-speaking refugees seems to be well-suited for the investigation of the relationship between stigma and the expression of somatic symptoms in depressed individuals.

Thus, the aim of the present study is to explore the representation of depressive symptoms in Arabic-speaking refugee outpatients. Furthermore, the prevalence of internalized mental health stigma will be assessed in order to investigate its relationship to the expression of psychological and somatic symptoms. Since various sources suspect that the bodily expression of distress is high in Arab cultures, *because* of prevalent mental health stigma (33–35), we test the hypothesis that internalized mental health stigma, which is the psychological impact of applying these negative societal stereotypes to oneself (42), is positively associated with the expression of somatic symptoms.

## Methods

### Participants

For the current cross-sectional study, a convenience sample of 100 Arabic-speaking refugees was recruited via the MEHIRA (Mental Health in Refugees and Asylum Seeker) study (43) between October 2018 and October 2019 in Berlin, Germany. Five individuals were excluded due to missing questionnaires, resulting in a total sample size of *N* = 95. An a priori power analysis indicated that a total sample size of *N* = 68 is required for the detection of a moderate effect (*f*^2^ = 0.15), with two predictors and a power of 80%, given an alpha error of 5%. Recruitment sites included the *Clearingstelle*, an outpatient facility for refugees in Berlin, and a psychiatric outpatient facility specialized in Arabic-speaking patients in Berlin, both established by the Charité Universitätsmedizin Berlin, Germany.

Inclusion criteria were defined as the following: (1) individuals between 18 and 65 years; (2) native Arabic speakers; (3) status of a refugee or asylum seeker which is defined according to the UNHCR as individuals who have been forced to flee their home country due to war, violence, or persecution (refugee) or as individuals who have requested sanctuary in another country and have applied for recognized refugee status there after fleeing their country (asylum seeker); who (4) show relevant symptoms of depression, defined by scoring “several days” or higher on the PHQ-9 on at least five of the nine items.

The exclusion criteria were: (1) missing informed consent; and a (2) current risk of suicidality based on clinical judgement or a score of four or more on the Montgomery–Åsberg Depression Rating Scale (MADRS) item 10.

### Procedure

Study participants were invited to take part in a baseline assessment, lasting ~90 min. Questionnaires of this comprehensive test-battery included, amongst others, the Arabic versions of the PHQ-15, the PHQ-9, the HTQ, and the ISMI, as well as socio-demographic information. All questionnaires were self-administered, yet an Arabic speaking psychologist surveilled the procedure and assisted in cases of illiteracy or need for further support. The data was then pseudonymized and transferred to a spreadsheet using the Statistical Package for the Social Sciences (SPSS) 22 for Windows (44). Since the study was conducted as a supplement to the MEHIRA study, it was covered and approved by the respective ethics vote issued from the ethics committee of the Charité – Universitätsmedizin Berlin (EA2/070/17).

### Questionnaires

#### Patient Health Questionnaire-15 (PHQ-15)

The PHQ-15 (45) is a brief and widely used self-administered screening instrument for the expression of somatic symptoms. Its 15 items cover over 90% of the physical symptoms seen in primary care, such as stomach/back pain and/or headaches, excluding upper respiratory tract symptoms. Participants indicate on a three-point Likert scale, how much they had been bothered by the respective symptom in the past 4 weeks, ranging from “not bothered at all” (0) to “bothered a lot” (2). Symptom severity can be classified according to a sum score, ranging from 0 to 30, while scores of ≥5, ≥10, ≥15 represent mild, moderate, and severe levels, respectively. The PHQ-15 has been proven to be highly reliable and valid in both clinical and occupational settings (45–48). Furthermore, it has been previously applied to screen for somatic symptoms across cultures and in refugee populations (49, 50). According to a review of 40 scales for the assessment of self-reported somatic symptoms, the PHQ-15 can be considered the best option for large-scale studies and cross-cultural comparisons based on several criteria including psychometric criteria, type of symptoms, time frame, languages, and patient burden (51). An Arabic translation of the PHQ-15 has been demonstrated to be both valid and highly reliable in a sample of Saudi University students, with a Cronbach's α of 0.83 (52). For the current study, Cronbach's α was 0.82.

#### Patient Health Questionnaire-9 (PHQ-9)

The PHQ-9 (53) is a self-administered diagnostic screening instrument for the assessment and monitoring of depression severity in primary care. The nine items of the scale assess each of the Diagnostic Criterion A symptoms for a Major Depressive Episode according to the DSM-IV (54). Participants indicate the degree to which they had been bothered by the respective symptom in the past 2 weeks on a four-point Likert scale, ranging from “not at all” (0) to “nearly every day” (3). The PHQ-9 sum-score can be divided into the following five categories of increasing symptom severity: minimal (2–9), mild (10–14), moderate (15–19), and severe (≥20). Furthermore, a cutoff-score of ≥10 has been recommended for the detection of a current Major Depression Episode (55). Numerous studies have demonstrated the validity and reliability of the PHQ-9 in specific medical populations, in the general population and psychiatric samples (46, 56–58). Furthermore, cross-cultural measurement invariance has been demonstrated for both the PHQ-9 and the PHQ-15 in two studies comparing Germans and migrants, indicating their applicability for cross-cultural research (59, 60). Two studies have been conducted to assess the reliability and validity of an Arabic translation of the PHQ-9 in Saudi University students (52) and a Lebanese outpatient sample (61). Evidence for factorial, discriminant, and convergent validity was provided, and the reliability of the scale was found to be high (0.86 ≤ α ≤ 0.88) (52, 61). In the present sample, Cronbach's α was 0.79.

#### The Brief Version of the Internalized Stigma of Mental Illness Scale (ISMI-10)

The ISMI-10 (62) is a brief, ten-item questionnaire for the assessment of internalized stigma of mental illness. In its original form, the ISMI comprises of 29 statements, measuring the five dimensions *alienation, discrimination experience, social withdrawal, stereotype endorsement*, and *stigma resistance* (63). The shortened version entails the two items of each subscale that demonstrated the strongest psychometric item qualities. Participants are asked to indicate their degree of agreement to a particular statement on a four-point Likert scale, ranging from “strongly disagree” (1) to “strongly agree” (4). The ISMI mean score can be interpreted following a 4-category method [minimal to no internalized stigma (1.00–2.00); mild (2.01–2.50); moderate (2.51–3.00); severe internalized stigma (3.01–4.00)] (64), or according to a 2-category method [does not report high internalized stigma (1.00–2.50); reports high internalized stigma (2.51–4.00)] (65). Comparable psychometric properties have been found for the ISMI-10 and the 29-item version, in terms of validity and reliability (62). In further validation studies, the scale was found to be reliable (0.75 ≤ α ≤ 0.86) and demonstrated predictive validity in relation to depression, physical health, self-esteem, functioning, recovery orientation, perceived devaluation and discrimination, empowerment, and quality of life (62, 66–68). To date, only the ISMI-29 has been translated into Arabic and validated within a refugee population (69). The Arabic version was shown to predict symptoms of depression, anxiety, and PTDS, and the reliability was found to be excellent (α = 0.94). For the present study, the 10 items of the ISMI-10 were selected out of the Arabic translation of the ISMI-29. Cronbach's α of this version was 0.70 in the current sample.

#### The Harvard Trauma Questionnaire (HTQ)

The HTQ (70) is the most widely used screening instrument for the assessment of trauma-related symptoms among refugee populations worldwide (71, 72). Part four covers 40 items related to PTSD and refugee-specific expressions of functional distress (73). The first 16 items of this last part are derived from the DSM-IV criteria for PTSD and are used for the purposes of the present study. Participants are asked to indicate on a four-point Likert scale how much they had been bothered by a respective symptom, ranging from “not at all” (1) to “extremely” (4). Individuals can be considered symptomatic for PTSD according to the DSM-IV if their mean score reaches the cut-off of ≥2.5. Across a wide range of populations, this measure has been found to be reliable, and convergent validity has been demonstrated (74). An Arabic translation of the 16 item measure of the HTQ by Shoeb et al. (75) was found to be highly reliable in a sample of Syrian Kurdish refugees, with a Cronbach's α of 0.88 (76). Furthermore, the number of instances of torture and other traumatic events experienced were positively related to PTSD symptoms, underlining the HTQ's concurrent validity (76). In the present study, Cronbach's α was 0.89.

### Statistical Analyses

All data was pseudonymized and stored in a password protected electronic spreadsheet. Statistical analyses were performed using the IBM Statistical Package for the Social Sciences (SPSS) 22 for Windows (44). Descriptive statistics were used to analyze the sample's socio-demographic characteristics (Table 1), as well as to provide an overview over the agreement to each individual item of the PHQ-15 and the PHQ-9 (Tables 2, 3). Multiple regression analyses were conducted with the PHQ-15 score and PHQ-9 score as dependent variables, and the ISMI and the HTQ as predictors to test for associations of stigma with somatic and psychological symptoms, while controlling for trauma (Table 4). Finally, an explanatory factor analysis was performed on the PHQ-15 items using varimax rotation to identify culture-specific symptom clusters (Table 5). The alpha level of significance was set at 5%.

**Table 1:** Sociodemographic characteristics of the survey sample.

**Sociodemographic data**	***N* = 95**
**Gender**	
Male	54 (56.8%)
Female	41 (43.2%)
**Age in years** M ± SD 33.80 ± 9.69^*^	
19–30	44 (46.3%)
31–40	29 (30.5%)
41–50	15 (15.8%)
51–64	7 (7.4%)
**Country of Origin**	
Syria	75 (78.9%)
Iraq	12 (12.6%)
Palestine	4 (4.2%)
Kuwait	1 (1.1%)
Jordan	1 (1.1%)
Lebanon	1 (1.1%)
Libya	1 (1.1%)
**Current state of residence**	
Permanent residence permit	2 (2.1%)
Temporary residence permit	84 (88.4%)
No legal residence permit	9 (9.5%)
**Education in years** M ± SD 10.46 ± 2.99^*^	
0–5	6 (6.3%)
6–10	28 (39.5%)
11–15	57 (60.0%)
>15	4 (4.2%)

* *Mean and Standard Deviation*.

**Table 2:** Mean and standard deviation for each item of the PHQ-15 and the total scale.

**PHQ-15**	***M (SD)***
**Total score (0–30)**	**13.24 (5.58)**
**Single items values**	
1. Stomach pain	0.69 (0.76)
2. **Back pain**	**1.26(0.75)**
3. Pain in your arms, legs, or joints	1.25 (0.76)
4. Menstrual cramps or other problems with your period (women only, *N* = 38)	1.03 (0.79)
5. Pain or problems during sexual intercourse	0.29 (0.54)
6. Headaches	1.25 (0.73)
7. Chest pain	0.85 (0.71)
8. Dizziness	0.79 (0.74)
9. Fainting spells	0.14 (0.37)
10. Feeling your heart pound or race	1.02 (0.68)
11. Shortness of breath	0.97 (0.78)
12. Constipation, loose bowels, or diarrhea	0.74 (0.75)
13. Nausea, gas, or indigestion	0.65 (0.74)
14. **Feeling tired or having low energy**	**1.53 (0.70)**
15. **Trouble sleeping**	**1.44 (0.74)**

*N = 95, the three items with the highest agreement are printed bold*.

**Table 3:** Mean and standard deviation for each item of the PHQ-9 and the total scale.

**PHQ-9**	***M (SD)***
**Total score (0–27)**	**16.28 (5.67)**
**Single items values**	
1. Little interest or pleasure in doing things	2.05 (0.92)
2. **Feeling down, depressed, or hopeless**	**2.18 (0.84)**
3. **Trouble falling or staying asleep, or sleeping too much**	**2.21 (1.01)**
4. **Feeling tired or having little energy**	**2.25 (0.89)**
5. Poor appetite or overeating	1.77 (1.17)
6. Feeling bad about yourself—or that you are a failure or have let yourself or your family down	1.65 (1.11)
7. Trouble concentrating on things, such as reading the newspaper or watching television	1.99 (1.05)
8. Moving or speaking so slowly that other people could have noticed? Or the opposite—being so fidgety or restless that you have been moving around a lot more than usual	1.26 (1.18)
9. Thoughts that you would be better off dead or of hurting yourself in some way	0.92 (1.09)

*N = 95, the three items with the highest agreement are printed bold*.

**Table 4:** Multiple regression analyses for the prediction of somatic and psychological symptoms by internalized stigma and the trauma.

	**PHQ-15^a^**				**PHQ-9^b^**		
**Variable**	***B***	***SE***	**β**		***B***	***SE***	**β**
ISMI	1.00	1.02	0.09		2.75	0.89	0.25^*^
HTQ	4.66	0.85	0.51^**^		5.08	0.72	0.57^**^
*R^2^*	0.29				0.47		
*F*	18.00^**^				38.93^**^		

a *N = 91*,

b *N = 92*;

** *p ≤ 0.001*,

* *p ≤ 0.01*.

**Table 5:** Explanatory factor analysis of the PHQ-15 items in Arabic-speaking refugees.

**Item**	**Factor loads**				
	**1**	**2**	**3**	**4**	**5**
Heart pound or race	**0.830**	0.028	0.057	0.123	−0.084
Shortness of breath	**0.784**	0.112	0.169	0.193	−0.117
Dizziness	**0.709**	0.236	−0.015	0.054	0.044
Chest pain	**0.588**	0.181	0.325	0.061	0.075
Painful sexual intercourse	−0.002	**0.677**	0.128	0.087	0.001
Tired/low energy	0.385	**0.540**	−0.022	0.301	0.051
Fainting spells	0.382	**0.496**	−0.219	−0.159	−0.030
Pain in arms, legs, joints	0.375	**0.484**	0.213	0.227	0.003
Constipation/diarrhea	−0.073	−0.198	**0.826**	0.141	0.045
Headaches	0.357	0.319	**0.608**	0.054	−0.018
Back pain	0.279	0.382	**0.593**	−0.027	−0.011
Stomach pain	0.042	0.227	−0.020	**0.859**	−0.088
Nausea, gas, indigestion	0.379	−0.050	0.264	**0.724**	0.112
Menstrual cramps	0.045	0.287	0.192	0.112	**0.826**
Trouble sleeping	0.145	0.381	0.195	0.161	**−0.738**
**Eigenvalues**	4.57	1.51	1.22	1.12	1.06
**% of variance**	30.48	10.07	8.16	7.49	7.09
**α**	0.78	0.56	0.55	0.68	−0.56

*The table shows the five extracted factors after principal factor extraction and varimax rotation with their initial eigenvalues, the percentage of explained variance and internal consistency by Arabic-speaking refugees. Factor loads of individual items >0.4 are printed bold. By logical grouping factors were defined as following: (1) symptoms of sadness, (2) pain-induced fatigue, (3) head-body related symptoms, (4) indigestion, and (5) male sleep problems*.

## Results

For the present study, the data of 95 participants, 54 males and 41 females, with a mean age of 33.80 years (*SD* = 9.69; range 19–64), were analyzed. With over 78%, the majority of the refugees in the sample named Syria as their country of origin, followed by Iraq (12.6%) and Palestine (4.2%). Only a small percentage of 2.1% received a permanent residence status from local authorities, whereas most individuals had a temporary residence status (88.4%), or even no legal residence permit (9.5%) to stay in Germany. On average, participants completed 10.46 years of schooling (*SD* = 2.99). Detailed information concerning all sociodemographic characteristics assessed is provided in Table 1. As such, the sociodemographic characteristics in terms of age and gender seem to be highly comparable to representative panel data of refugees living in Germany (77). In terms of education, a comparison is rather difficult due to the different types of assessment (years of education vs. international standard of education). Yet, the samples also seem comparable in this respect.

### Symptom Representation

Descriptive analyses were performed for both the PHQ-15 and the PHQ-9 to explore the expression of depressive symptoms in the present sample. The results are depicted in Table 2 for the PHQ-15 and Table 3 for the PHQ-9. For the PHQ-15, the calculated mean score of 13.24 (*SD* = 5.58) indicates a moderate level of somatic symptoms. A detailed analysis on the item level revealed that the Arabic-speaking refugees in the sample were mostly bothered by *feeling tired or having low energy* (*M* = 1.53; *SD* = 0.70), followed by *trouble sleeping* (*M* = 1.44; *SD* = 0.74), and *back pain* (*M* = 1.26; *SD* = 0.75). For the PHQ-9, the mean score of 16.28 (*SD* = 5.67) is also indicative of a moderate level of psychological symptoms. Here, the symptoms that were experienced most frequently by the participants were *feeling tired or having little energy* (*M* = 2.25; *SD* = 0.89), followed by *trouble falling or staying asleep, or sleeping too much* (*M* = 2.21; *SD* = 1.01), and *feeling down, depressed, or hopeless* (*M* = 2.18; *SD* = 0.84). A correlation analysis revealed a moderately significant positive association between psychological and somatic symptoms (*r* = 0.54, *p* < 0.001).

### Influence of Stigma on Symptom Representation

For the following analyses, three participants had to be excluded because they did not provide any answer to the ISMI scale. On average, participants displayed a rather low level of internalized stigma (*M* = 2.25; *SD* = 0.50), corresponding to *mild internalized stigma* according to the 4-category method, or to the category of *does not report high internalized stigma* according to the 2-category method (see above). The mean score of 2.58 (*SD* = 0.61) in the HTQ shows that, on average, individuals of the sample show relevant symptoms of PTSD according to the DSM-IV.

These two variables were entered as predictors into multiple regression analyses with the PHQ-15 and the PHQ-9 as dependent variables to test for associations of stigma with somatic and psychological symptoms while controlling for the well-established association of trauma and the expression of somatic symptoms (78) (Table 4). For the regression model with the PHQ-15 as the dependent variable, one further participant had to be excluded, since his/her studentized deleted residual of 3.19 was classified as an outlier. No participant was excluded following a regression diagnostics procedure for the model with the PHQ-9 as a dependent variable.

For the PHQ-15, results revealed that the HTQ was the only significant predictor for the PHQ-15 score (β = 0.51*, p* < 0.001), whereas the ISMI did not reach statistical significance (β = 0.09*, p* = 0.16). In total, this model could explain 29% of the variance in the PHQ-15 score (*F*_(2, 88)_ = 18.00, *p* < 0.001). For the PHQ-9, significant positive associations were found with both the ISMI (β = 0.25*, p* = 0.002) and the HTQ (β = 0.57*, p* < 0.001). Together, these two predictors accounted for 47% of the variance in the PHQ-9 score (*F*_(2, 89)_ = 38.93, *p* < 0.001). These results do not support the postulated hypothesis that internalized stigma is associated with more somatic symptom expression.

### Factor Structure of the PHQ-15 in a Sample of Arabic Speaking Refugees

Furthermore, the factor structure of all PHQ-15 items was examined in the present sample. The Kaiser-Meyer-Olkin measure of sampling adequacy was 0.724, which is above the recommended value of 0.5. Bartlett's test of sphericity was significant (χ^2^(105) = 334.36, *p* < 0.001). A principal factor extraction was performed using varimax rotation. The rotated factor matrix is depicted in Table 5. In this way, five distinct factors could be identified and were labeled with the help of an Arabic psychologist: (1) symptoms of sadness, (2) pain-induced fatigue, (3) head-body-related symptoms, (4) indigestion, and (5) male sleep problems.

### Missing Values

Lastly, a close inspection of missing values was conducted to identify any regularities. Generally, missing values were rare and occurred only in two items of the PHQ-15: The item *Menstrual cramps or other problems with your period* was not answered by three (female) participants, and the item *Pain or problems during sexual intercourse* was left unanswered by 16 participants. Of the latter ones, 14 participants identified as male.

## Discussion

The present study aimed to explore the representation of depressive symptoms in a sample of Arabic-speaking refugee outpatients. Specifically, the expression of psychological and somatic distress was analyzed to inform clinicians about the most prevalent symptoms occurring within the largest refugee population in Germany. Furthermore, the prevalence of internalized stigma was examined to empirically investigate the supposed relationship between stigma and somatic symptom expression. The main results of this research show that Arabic-speaking refugee outpatients express a moderate level of both somatic and psychological symptoms of depression, and that stigma does not seem to be associated with somatic symptoms, but rather with psychological symptoms.

The present findings add empirical evidence from a rather under-researched population to the debate about somatization and psychologization across cultures. The moderate level of somatic symptoms expressed supports clinical observational data, which have reported a high level of bodily distress in Arab mental health patients and refugees (34, 79). This is even substantiated by an in-depth analysis of single items of the PHQ-9 which shows that the most prevalent psychological symptoms were the ones that were shared and overlap with the PHQ-15, namely a “feeling of energy loss” and “sleep disturbances.” Therefore, these key depressive symptoms can be considered at least quasi-somatic, which highlights the role of somatic symptoms in Arabic-speaking refugees. Nevertheless, the rate of psychological depressive symptoms was also found to be substantial, and a moderately positive correlation was found between psychological and somatic symptoms. Even when the two overlapping items were deleted from both scales, the strength of the relationship still approached a significant, moderately positive level of association (*r* = 0.46, *p* < 0.001). As such, these findings are in line with the ones obtained by Lee et al. (24), who found that psychological and somatic distress coexisted in their Chinese population-based sample. Therefore, this study can be understood as another challenge for the Western mind-body dualism, as it shows that the experience of somatic distress does not preclude the simultaneous experience of psychological symptoms (26, 80).

The high prevalence of sleep problems mirrored in both the PHQ-9 and the PHQ-15 is not surprising, given that they are considered a core symptom of both depression and PTSD (81). As such, these findings are consistent with those by Sandahl et al. (82), who report that about 99% of their sample of 752 traumatized refugees reported having trouble sleeping and recurrent nightmares. Furthermore, a growing body of research shows that sleep disturbances in the context of depression have been linked to an increased risk of adverse health outcomes, including, functional impairment, an increased risk for suicidality, non-remittance, as well as decrements in mobility, self-care, cognition, pain, and interpersonal activities [for an overview see Stickley et al. (83)]. Thus, these findings have important implications for clinicians working with Arabic-speaking refugees, since interventions to improve sleep quality might have the potential to alleviate their psychological distress (81). A recently published manual for group therapy sessions with refugees can serve as a valuable source in this respect (84).

A further goal of the present research was the investigation of potential associations of internalized stigma with the representation of depressive symptoms. Contrary to the postulated hypothesis, our results show no association between stigma and somatic symptoms and thus provide no evidence for the supposed association between these constructs in the literature concerning Arab mental health (33–35). Moreover, these findings contribute to the open debate on the relation of stigma and the expression of bodily distress in cross-cultural research and substantiate evidence from previous research that did not find such associations (31, 32). However, stigma was found to be related to the severity of psychological symptoms when trauma symptoms were controlled. Various sources have reported similar findings, yet, due to the cross-sectional study design of this and previous studies, no causal relationship can be inferred (69, 85, 86). This highlights the need for further experimental studies that seek to lower stigma and investigate whether the depression severity level can be effectively reduced through such interventions.

Interestingly, the level of internalized stigma was relatively low in the present sample. This was unexpected, given that previous research has quite consistently demonstrated a high prevalence of mental health stigma in both Arab cultures and refugee populations (36–40). In a recent study, Karnouk et al. (87) report a similarly low level of stigma in psychiatric patients from the Jordanian host- and refugee community. As a possible explanation, the authors argue that this decrease might represent the effect of current efforts in the Arab world to meet the need for mental health care services and to raise public awareness of mental health issues (88). Alternatively, this low level of stigma might be the result of sampling bias. The current convenience sample comprised of treatment seeking individuals, who voluntarily participated in a study on refugee mental health care. As such, it is to be expected that these individuals generally have lower stigma concerning mental health issues compared to the ones who denied their participation or did not seek treatment at all. Moreover, the at least basic education level, as well as the rather young mean age of the sample, might have contributed to the low level of stigma observed (89, 90). In fact, a *post-hoc* correlation analysis revealed a positive association between age and stigma (*r* = 0.27, *p* = 0.010), yet, no association between years of schooling and stigma was found (*r* = −0.10, *p* = 0.353). However, given the restricted variance in the sociodemographic variables in the sample, these results are tentative at best and such analyses are recommend for future research with a more divers sample. Given that the demographic characteristics are similar to the ones obtained in by a representative panel study of refugees living in Germany (77), the present results concerning the low level of stigma might be transferable to the population of Arabic-speaking refugees in Germany, at least in this respect.

The explanatory approach for the identification of specific symptom clusters resulted in five independent factors that were named with the help of an Arabic psychologist: (1) symptoms of sadness, (2) pain-induced fatigue, (3) head-body related symptoms, (4) indigestion, and (5) male sleep problems. Three of these clusters have also been identified in a qualitative study with four focus groups within the Arab community in Dubai, who sought to identify the terms and descriptions that are commonly used for depressive symptoms (91). The first factor of symptoms of sadness is similar to the description of “[a] feeling of tightness or constriction in the chest […where] the depressed person feels unable to take a deep breath [… because] the chest is felt to be too tightly packed with an excess of unpleasant feelings […]” (p. 216). The second factor of pain-induced fatigue is described as “[f]atigue due to generalized aches [… with] a subjective feeling of lack of body energy and soundness (ta'bana), the limbs suffering the most” (p. 216). It has to be noted that the high loading of the item painful sexual intercourse has to be interpreted with caution due to the observed missing values. Lastly, the fourth factor of indigestion resembles the cluster of “[a]limentary symptoms in the form of nausea or sickness and poor appetite, which are attributed to the abdomen and particularly to the liver (chabid)” (p. 216).

Whereas, these three factors provide empirical evidence for clusters that have been identified by previous qualitative research (91), the interpretation of the factors three and five seems to be less straightforward. With constipation or diarrhea, headaches, and back pain, factor three comprises somatic symptoms from very different locations in the body. According to Hassan et al. such pain sensations in different body parts including “[…] cramps in the guts, or pain in the stomach or in the head […]” (p. 23) have been found to be a typical expression of fatigue and general distress in war-affected Syrians, coupled with a perception that the organs are unable to contain the distress (92). Factor five combines the items menstrual cramps and trouble sleeping with inverse factor loadings. Since male individuals had a mean score of 0 on the item menstrual cramps, the inverse association was assumed to be indicative of a higher severity level of sleep problems in men than in women. A *t*-test supported this assumption (*t*(72.22) = −2.24, *p* = 0.03). This is interesting, given that females have been previously found to exhibit more sleep problems compared to men (93). Further studies are necessary to investigate whether this observation describes a pattern in Arabic-speaking refugees. Also, it is suggested to perform individual factor analyses on the PHQ-15 items for males and females in studies with larger sample sizes.

These symptom clusters highlight the reciprocal relationship of explanatory models of mental illness with language and culture. In Arabic, emotions are usually described with metaphors and imagery drawn from rich poetic cultural resources (34, 92, 94). Especially references to the heart seem common for the description of depressive symptoms and distress. Hassan et al. (92) have compiled a list of commonly used expressions and idioms for distress in Syrian Arabic on the basis of suggestions by various Arabic-speaking mental health professionals. Depressive symptoms are described by a feeling of “heaviness in the heart,” “pain in the heart,” or a “squeezed heart,” or by phrases such as “blindness got to my heart” and “my heart is broken” (p. 23–26). Thus, it is not surprising that the first factor, comprising symptoms such as a pounding heart, shortness of breath, and chest pain, could explain the highest proportion of the variance in all somatic symptoms. A better understanding of such idioms might enhance a clinical conversation with Arabic-speaking mental health patients and could even inform interventions and treatment approaches (92).

The analysis of missing values revealed that specifically shame related items, including menstrual cramps and painful sexual intercourse, were occasionally left unanswered by participants. Especially males did not answer the item on sexual pain and some explained this with the absence of their wife. Even though this answer seems plausible, the pattern observed might additionally point to an often documented, still prevailing taboo of sexuality-related issues in the Arab world, as well as a lack of education on these matters (95, 96). Thus, the present findings might argue for special care and cultural awareness when talking about sexuality-related topics with Arabic-speaking refugees in research or health care settings. Therefore, it is recommended to match patients with clinicians or interviewers of the same sex, at least at the beginning of therapy, a procedure that could not always be assured in the present research concerning the surveilling psychologist due to limited resources (97).

A particular strength of the present study lies in the selection of measures for somatic and psychological symptoms of depression that have been explicitly recommended for cross-cultural research (51). This lays the foundation for the comparison of the given results with results from studies with refugee populations from other cultural backgrounds and thus satisfies a call by Rohlof et al. (79), who criticized that the abundance of different, often non-validated measures exacerbate a global understanding of bodily distress in refugees.

The present results have to be interpreted in light of several limitations: Firstly, concerning the sample recruited the selection bias has resulted in a convenience sample that might not be representative of the population of Arabic-speaking refugees with symptoms of depression, especially concerning the level of mental health stigma. Furthermore, the convenience sample consisted of refugees from mostly Syrian descent, which might impede the generalizability to Arabic-speaking refugees in general and should thus be considered in future research. However, since the vast majority of refugees worldwide as well as in Germany originate from Syria, the sample seems representative for the underlying population. It is also highly recommended to recruit a larger sample in future research in order to analyze how variables like gender or age moderate the associations observed. Secondly, depressive symptoms were merely assessed with the PHQ-9, since clinical diagnoses were not available for all participants. It is recommended to use standardized clinical interviews or expert diagnoses in further studies to specifically investigate symptoms of Arabic-speaking refugees with a diagnosis of depression. Likewise, information concerning comorbidities, medication, or other demographic variables like residence time in Germany could not be included here, but would be vital in future research. Thirdly, the use of self-report questionnaires might have resulted in common method variance, which might have increased the observed effects in the regression analyses. Yet, it has to be noted that self-report questionnaires might be especially appropriate for research in this population, since this method has been found to reduce a respondent's discomfort and embarrassment for sensitive issues and might thus result in more reliable data (98). Concerning the questionnaires used, it also has to be mentioned that the only the ISMI-29, but not the ISMI-10 used, has been validated in an Arabic-speaking refugee population and future research is encouraged to use validated questionnaires to minimize measurement bias. Fourthly, no comparison group was included from another cultural background. This would have been especially necessary for the analysis and interpretation of the symptom clusters found in order to infer culture-specific regularities. Therefore, the inclusion of comparison groups is highly recommended for further research. Lastly, it needs to be stressed that even though country of origin was not used as a proxy for culture, individuals from very different backgrounds were collapsed into the category of Arabic-speaking refugees for the purpose of the present study. As suggested by Kirmayer and Ryder (21), this grouping was based on specific cultural contexts and processes such as shared language background and experiences of flight, nevertheless, this leads to an impression of a rather homogenous group which is certainly not the case.

In conclusion this study provides empirical evidence that both somatic and psychological symptoms are commonly used forms of expressing depressive symptoms in Arabic-speaking refugees, while problems with sleep and energy loss seem to be the most prevalent symptoms reported. Although these results should be interpreted with caution, it does not appear that a higher level of somatic symptom expression can be traced back to mental health stigma, but rather to culture-specific explanatory models, idioms, and expressions. The implications that arise from these findings are that mental health professionals should be trained more thoroughly in both the special mental health needs of Arabic-speaking refugees as well as in culturally mediated modes of symptom interpretation and expression. Given that refugees in Germany seldomly receive adequate mental health treatment (10), learning about typical symptoms and cultural codes might help improve our understanding of a cultural barrier, i.e., the way of expressing depressive symptoms, and might eventually contribute to faster diagnosis and better mental health care provision for the largest refugee population in Germany.

## Data Availability Statement

The raw data supporting the conclusions of this article will be made available by the authors, without undue reservation.

## Ethics Statement

The studies involving human participants were reviewed and approved by Ethical Committee of Charité - Universitätsmedizin Berlin (EA2/070/17). The patients/participants provided their written informed consent to participate in this study.

## Author Contributions

NL contributed to the conceptualization of the idea, data analysis and interpretation, as well as to writing. CK contributed to scale selection, data collection logistics, interpretation of the data, and supervision. EH, MB, and KB contributed to the conceptualization of the idea and study design, revision, and supervision. DR, DC, and LS contributed to scale selection and translation, as well as to data collection logistics. All authors read and approved the final manuscript.

## Conflict of Interest

The authors declare that the research was conducted in the absence of any commercial or financial relationships that could be construed as a potential conflict of interest.
